# (*E*)-3-(3,4-Dimethoxy­phen­yl)-1-(2-fur­yl)prop-2-en-1-one

**DOI:** 10.1107/S1600536808020539

**Published:** 2008-07-09

**Authors:** Hoong-Kun Fun, P. S. Patil, Samuel Robinson Jebas, S. M. Dharmaprakash

**Affiliations:** a X-ray Crystallography Unit, School of Physics, Universiti Sains Malaysia, 11800 USM, Penang, Malaysia; b Department of Studies in Physics, Mangalore University, Mangalagangotri, Mangalore 574 199, India

## Abstract

In the title mol­ecule, C_15_H_14_O_4_, the benzene and furyl rings are inclined to each other with a dihedral angle of 41.5 (1)°. An intra­molecular C—H⋯O hydrogen-bond inter­action generates an *S*(5) ring motif. In the crystal structure, mol­ecules are stacked along the *b* axis and the crystal packing is stabilized by inter­molecular C—H⋯O and C—H⋯π inter­actions. In addition, π–π stacking inter­actions with a centroid-to-centroid distance of 3.5855 (11) Å are observed.

## Related literature

For related literature on the non-linear optical properties of chromophore derivatives, see: Agrinskaya *et al.* (1999 ▶). For other related literature, see: Chantrapromma *et al.* (2005 ▶, 2006 ▶); Fun *et al.* (2006 ▶); Patil, Fun *et al.* (2007 ▶); Patil, Dharmaprakash *et al.* (2007 ▶); Patil *et al.* (2006 ▶). For bond-length data, see: Allen *et al.* (1987 ▶). For graph-set analysis of hydrogen bonding, see: Bernstein *et al.* (1995 ▶).
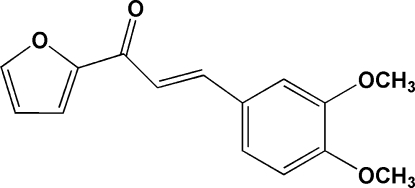


## Experimental

### 

#### Crystal data

C_15_H_14_O_4_
*M* *_r_* = 258.26
Monoclinic, 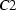

*a* = 21.5582 (5) Å
*b* = 5.6105 (1) Å
*c* = 10.4622 (3) Å
β = 101.510 (2)°
*V* = 1239.98 (5) Å^3^
*Z* = 4
Mo *K*α radiation
μ = 0.10 mm^−1^
*T* = 100.0 (1) K
0.42 × 0.05 × 0.04 mm

#### Data collection

Bruker APEXII CCD area-detector diffractometer
Absorption correction: multi-scan (*SADABS*; Bruker, 2005 ▶) *T*_min_ = 0.947, *T*_max_ = 0.996
7017 measured reflections
1997 independent reflections
1707 reflections with *I* > 2σ(*I*)
*R*_int_ = 0.033

#### Refinement

*R*[*F* ^2^ > 2σ(*F* ^2^)] = 0.042
*wR*(*F* ^2^) = 0.104
*S* = 1.10
1997 reflections
174 parameters
1 restraint
H-atom parameters constrained
Δρ_max_ = 0.29 e Å^−3^
Δρ_min_ = −0.20 e Å^−3^

### 

Data collection: *APEX2* (Bruker, 2005 ▶); cell refinement: *APEX2*; data reduction: *SAINT* (Bruker, 2005 ▶); program(s) used to solve structure: *SHELXTL* (Sheldrick, 2008 ▶); program(s) used to refine structure: *SHELXTL*; molecular graphics: *SHELXTL*; software used to prepare material for publication: *SHELXTL* and *PLATON* (Spek, 2003 ▶).

## Supplementary Material

Crystal structure: contains datablocks global, I. DOI: 10.1107/S1600536808020539/lh2655sup1.cif

Structure factors: contains datablocks I. DOI: 10.1107/S1600536808020539/lh2655Isup2.hkl

Additional supplementary materials:  crystallographic information; 3D view; checkCIF report

## Figures and Tables

**Table 1:** Hydrogen-bond geometry (Å, °) *Cg*1 is the centroid of the ring C8–C13

*D*—H⋯*A*	*D*—H	H⋯*A*	*D*⋯*A*	*D*—H⋯*A*
C1—H1*A*⋯O2^i^	0.93	2.32	3.247 (3)	173
C3—H3*A*⋯O3^ii^	0.93	2.42	3.338 (3)	167
C7—H7*A*⋯O2	0.93	2.47	2.810 (3)	101
C14—H14*B*⋯*Cg*1^iii^	0.96	2.66	3.431 (2)	137

Symmetry codes: (i) 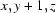; (ii) 
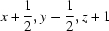
; (iii) 
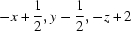
.

**Fig. 1.**
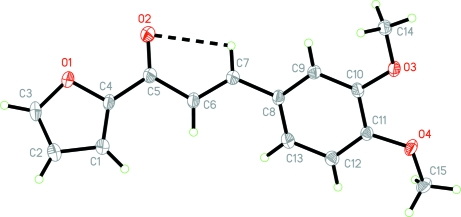
The molecular structure of the title compound, showing 50% probability displacement ellipsoids and the atom numbering scheme. The dashed line indicates a hydrogen bond.

**Fig. 2.**
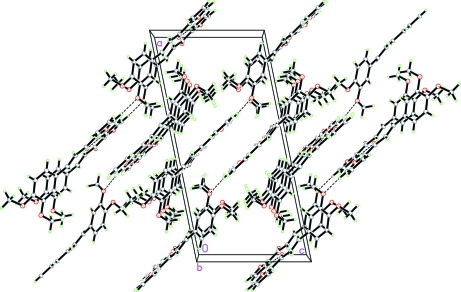
The crystal packing of the title compound, viewed along the b axis. Hydrogen bonds are shown as dashed lines.

## supplementary crystallographic information

### Comment

Among the many types of NLO chromophores developed so far, the dipolar push–pull molecules consisting of electron donor and acceptor groups inter-bridged by a π-segment have received the predominant attention (Agrinskaya *et al.*, 1999). As a part of the investigation of nonlinear compounds (Chantrapromma *et al.*, 2005, 2006; Fun *et al.*, 2006; Patil, Fun *et al.*, 2007; Patil, Dharmaprakash *et al.*, 2007; Patil *et al.*, 2006), the title compound (I) has recently been prepared in our laboratory and its crystal structure is presented here. The non-centrosymmetric crystal of the title compound should exhibit second-order NLO properties.

The bond lengths and bond angles in (I)  have normal values (Allen *et al.*, 1987). The benzene and furyl rings in the molecule are essentially planar with the maximum deviation from planarity being 0.016 (2) Å for atom C10 and 0.003 (2) Å for atom C4 respectively. The dihedral angle between the phenyl and the furyl rings is 41.5 (1)°, indicating that they are twisted from each other.

An intramolecular C—H···O hydrogen bond generates a S(5) ring motif (Bernstein *et al.*, 1995). In the crystal structure, the molecules are stacked along the *b* axis. The crystal packing is consolidated by C—H···O and C—H···π interactions. π–π interactions with the centroid···centroid(1 -*x*, *y*,3 - *z*) distance of 3.5855 (11) Å is observed.

### Experimental

3,4-dimethoxybenzaldehyde (0.01 mol, 1.66 g m) in ethanol (20 ml) was mixed with 2-acetyl furan (0.01 mol, 1.01 ml) in 20 ml ethanol and the mixture was treated with 5 ml of 10% sodium hydroxide solution and stirred at room temperature for 6 h. The precipitate obtained was poured into ice-cold water (500 ml) and left to stand for 5 h. The resulting crude solid was filtered, dried and recrystallized from N,*N*-dimethylformamide (DMF) by slow evaporation.

### Refinement

H atoms were positioned geometrically [C—H = 0.93Å and 0.96 Å for methyl H atoms] and refined using a riding model, with *U*_iso_(H) = 1.2*U*_eq_(C) and 1.5U_equ_(C) methyl. The rotating group model was used for the methyl group hydrogen atoms. In the absence of significant anomalous dispersion effects 1283 Friedel pairs were merged.

### Crystal data

**Table:**

C_15_H_14_O_4_	*F*_000_ = 544
*M**_r_* = 258.26	*D*_x_ = 1.383 Mg m^−^^3^
Monoclinic, *C*2	Mo *K*α radiation λ = 0.71073 Å
Hall symbol: C 2y	Cell parameters from 1982 reflections
*a* = 21.5582 (5) Å	θ = 3.0–30.0º
*b* = 5.6105 (1) Å	µ = 0.10 mm^−^^1^
*c* = 10.4622 (3) Å	*T* = 100.0 (1) K
β = 101.510 (2)º	Needle, colourless
*V* = 1239.98 (5) Å^3^	0.42 × 0.05 × 0.04 mm
*Z* = 4	

### Data collection

**Table:**

Bruker APEXII CCD area-detector diffractometer	1997 independent reflections
Radiation source: fine-focus sealed tube	1707 reflections with *I* > 2σ(*I*)
Monochromator: graphite	*R*_int_ = 0.033
*T* = 100.0(1) K	θ_max_ = 30.2º
φ and ω scans	θ_min_ = 1.9º
Absorption correction: multi-scan(SADABS; Bruker, 2005)	*h* = −30→30
*T*_min_ = 0.947, *T*_max_ = 0.996	*k* = −7→7
7017 measured reflections	*l* = −14→14

### Refinement

**Table:**

Refinement on *F*^2^	Secondary atom site location: difference Fourier map
Least-squares matrix: full	Hydrogen site location: inferred from neighbouring sites
*R*[*F*^2^ > 2σ(*F*^2^)] = 0.042	H-atom parameters constrained
*wR*(*F*^2^) = 0.104	*w* = 1/[σ^2^(*F*_o_^2^) + (0.059*P*)^2^] where *P* = (*F*_o_^2^ + 2*F*_c_^2^)/3
*S* = 1.10	(Δ/σ)_max_ < 0.001
1997 reflections	Δρ_max_ = 0.29 e Å^−^^3^
174 parameters	Δρ_min_ = −0.20 e Å^−^^3^
1 restraint	Extinction correction: none
Primary atom site location: structure-invariant direct methods	

### Special details

**Table:**

Experimental. The data was collected with the Oxford Cyrosystem Cobra low-temperature attachment.
Geometry. All e.s.d.'s (except the e.s.d. in the dihedral angle between two l.s. planes) are estimated using the full covariance matrix. The cell e.s.d.'s are taken into account individually in the estimation of e.s.d.'s in distances, angles and torsion angles; correlations between e.s.d.'s in cell parameters are only used when they are defined by crystal symmetry. An approximate (isotropic) treatment of cell e.s.d.'s is used for estimating e.s.d.'s involving l.s. planes.
Refinement. Refinement of *F*^2^ against ALL reflections. The weighted *R*-factor *wR* and goodness of fit *S* are based on *F*^2^, conventional *R*-factors *R* are based on *F*, with *F* set to zero for negative *F*^2^. The threshold expression of *F*^2^ > σ(*F*^2^) is used only for calculating *R*-factors(gt) *etc*. and is not relevant to the choice of reflections for refinement. *R*-factors based on *F*^2^ are statistically about twice as large as those based on *F*, and *R*- factors based on ALL data will be even larger.

### Fractional atomic coordinates and isotropic or equivalent isotropic displacement parameters (Å^2^)

**Table:**

	*x*	*y*	*z*	*U*_iso_*/*U*_eq_	
O1	0.57239 (6)	0.0466 (3)	1.44832 (13)	0.0232 (4)	
O2	0.48812 (8)	−0.1128 (3)	1.23361 (16)	0.0332 (4)	
O3	0.20877 (6)	0.3835 (3)	0.77192 (13)	0.0207 (3)	
O4	0.25511 (6)	0.7519 (3)	0.67784 (12)	0.0205 (3)	
C1	0.55488 (9)	0.4315 (4)	1.39689 (18)	0.0214 (5)	
H1A	0.5388	0.5703	1.3540	0.026*	
C2	0.60096 (9)	0.4149 (5)	1.51546 (19)	0.0243 (5)	
H2A	0.6211	0.5408	1.5648	0.029*	
C3	0.60942 (8)	0.1816 (5)	1.54141 (19)	0.0235 (5)	
H3A	0.6370	0.1201	1.6137	0.028*	
C4	0.53928 (8)	0.2049 (4)	1.35948 (17)	0.0186 (4)	
C5	0.49518 (9)	0.1039 (4)	1.2480 (2)	0.0208 (4)	
C6	0.46005 (8)	0.2776 (4)	1.15509 (17)	0.0195 (4)	
H6A	0.4711	0.4380	1.1614	0.023*	
C7	0.41218 (8)	0.2036 (4)	1.06159 (18)	0.0197 (4)	
H7A	0.4025	0.0420	1.0601	0.024*	
C8	0.37371 (8)	0.3532 (4)	0.96152 (17)	0.0177 (4)	
C9	0.31001 (8)	0.2882 (4)	0.91378 (17)	0.0178 (4)	
H9A	0.2939	0.1504	0.9443	0.021*	
C10	0.27144 (8)	0.4282 (4)	0.82177 (17)	0.0178 (4)	
C11	0.29632 (8)	0.6304 (4)	0.77127 (17)	0.0164 (4)	
C12	0.35917 (8)	0.6949 (4)	0.81736 (18)	0.0189 (4)	
H12A	0.3758	0.8294	0.7844	0.023*	
C13	0.39718 (8)	0.5570 (4)	0.91321 (18)	0.0189 (4)	
H13A	0.4389	0.6026	0.9451	0.023*	
C14	0.18004 (8)	0.1967 (4)	0.83195 (19)	0.0209 (4)	
H14A	0.1353	0.1960	0.7980	0.031*	
H14B	0.1878	0.2219	0.9245	0.031*	
H14C	0.1977	0.0465	0.8138	0.031*	
C15	0.28193 (10)	0.9223 (5)	0.60272 (19)	0.0232 (4)	
H15A	0.2497	0.9791	0.5324	0.035*	
H15B	0.3152	0.8484	0.5679	0.035*	
H15C	0.2989	1.0537	0.6574	0.035*	

### Atomic displacement parameters (Å^2^)

**Table:**

	*U*^11^	*U*^22^	*U*^33^	*U*^12^	*U*^13^	*U*^23^
O1	0.0204 (6)	0.0220 (9)	0.0236 (7)	0.0022 (6)	−0.0043 (5)	0.0049 (7)
O2	0.0346 (8)	0.0191 (9)	0.0376 (9)	0.0034 (7)	−0.0128 (7)	0.0013 (9)
O3	0.0168 (5)	0.0235 (9)	0.0198 (6)	−0.0018 (6)	−0.0014 (5)	0.0065 (7)
O4	0.0204 (6)	0.0228 (9)	0.0175 (6)	0.0022 (6)	0.0015 (5)	0.0081 (7)
C1	0.0210 (8)	0.0222 (13)	0.0196 (8)	0.0002 (8)	0.0003 (7)	0.0021 (9)
C2	0.0227 (8)	0.0295 (14)	0.0187 (8)	−0.0004 (9)	−0.0009 (7)	−0.0014 (10)
C3	0.0172 (8)	0.0337 (15)	0.0174 (8)	0.0027 (9)	−0.0021 (6)	0.0028 (10)
C4	0.0158 (7)	0.0211 (11)	0.0173 (8)	0.0028 (8)	−0.0005 (6)	0.0039 (9)
C5	0.0190 (8)	0.0190 (12)	0.0224 (9)	0.0030 (8)	−0.0005 (7)	0.0003 (9)
C6	0.0187 (8)	0.0201 (11)	0.0176 (8)	0.0029 (8)	−0.0012 (6)	0.0009 (9)
C7	0.0193 (8)	0.0173 (11)	0.0205 (9)	0.0012 (8)	−0.0008 (7)	0.0006 (9)
C8	0.0176 (7)	0.0179 (11)	0.0161 (8)	0.0043 (8)	−0.0002 (6)	−0.0015 (9)
C9	0.0197 (8)	0.0159 (11)	0.0168 (8)	0.0014 (7)	0.0015 (6)	−0.0006 (8)
C10	0.0165 (7)	0.0197 (11)	0.0158 (8)	0.0008 (7)	−0.0001 (6)	−0.0015 (9)
C11	0.0182 (8)	0.0169 (11)	0.0133 (8)	0.0021 (7)	0.0015 (6)	−0.0010 (8)
C12	0.0197 (8)	0.0204 (12)	0.0167 (8)	−0.0008 (8)	0.0033 (6)	0.0007 (9)
C13	0.0158 (7)	0.0224 (12)	0.0178 (8)	0.0012 (8)	0.0016 (6)	−0.0026 (9)
C14	0.0189 (8)	0.0234 (12)	0.0194 (8)	−0.0022 (8)	0.0016 (6)	0.0026 (9)
C15	0.0305 (9)	0.0196 (12)	0.0189 (8)	0.0003 (9)	0.0033 (7)	0.0063 (9)

### Geometric parameters (Å, °)

**Table:**

O1—C3	1.360 (3)	C7—C8	1.464 (3)
O1—C4	1.377 (2)	C7—H7A	0.9300
O2—C5	1.231 (3)	C8—C13	1.386 (3)
O3—C10	1.371 (2)	C8—C9	1.412 (2)
O3—C14	1.425 (3)	C9—C10	1.384 (3)
O4—C11	1.365 (2)	C9—H9A	0.9300
O4—C15	1.431 (3)	C10—C11	1.402 (3)
C1—C4	1.353 (3)	C11—C12	1.392 (2)
C1—C2	1.429 (2)	C12—C13	1.396 (3)
C1—H1A	0.9300	C12—H12A	0.9300
C2—C3	1.342 (4)	C13—H13A	0.9300
C2—H2A	0.9300	C14—H14A	0.9600
C3—H3A	0.9300	C14—H14B	0.9600
C4—C5	1.464 (3)	C14—H14C	0.9600
C5—C6	1.475 (3)	C15—H15A	0.9600
C6—C7	1.339 (2)	C15—H15B	0.9600
C6—H6A	0.9300	C15—H15C	0.9600
			
C3—O1—C4	105.99 (18)	C10—C9—C8	120.4 (2)
C10—O3—C14	116.67 (15)	C10—C9—H9A	119.8
C11—O4—C15	116.78 (14)	C8—C9—H9A	119.8
C4—C1—C2	106.2 (2)	O3—C10—C9	124.77 (19)
C4—C1—H1A	126.9	O3—C10—C11	115.27 (16)
C2—C1—H1A	126.9	C9—C10—C11	119.95 (16)
C3—C2—C1	106.4 (2)	O4—C11—C12	124.57 (19)
C3—C2—H2A	126.8	O4—C11—C10	115.50 (15)
C1—C2—H2A	126.8	C12—C11—C10	119.92 (17)
C2—C3—O1	111.20 (17)	C11—C12—C13	119.73 (19)
C2—C3—H3A	124.4	C11—C12—H12A	120.1
O1—C3—H3A	124.4	C13—C12—H12A	120.1
C1—C4—O1	110.20 (16)	C8—C13—C12	120.98 (16)
C1—C4—C5	132.75 (19)	C8—C13—H13A	119.5
O1—C4—C5	117.05 (19)	C12—C13—H13A	119.5
O2—C5—C4	121.66 (19)	O3—C14—H14A	109.5
O2—C5—C6	122.51 (18)	O3—C14—H14B	109.5
C4—C5—C6	115.8 (2)	H14A—C14—H14B	109.5
C7—C6—C5	119.7 (2)	O3—C14—H14C	109.5
C7—C6—H6A	120.1	H14A—C14—H14C	109.5
C5—C6—H6A	120.1	H14B—C14—H14C	109.5
C6—C7—C8	126.1 (2)	O4—C15—H15A	109.5
C6—C7—H7A	117.0	O4—C15—H15B	109.5
C8—C7—H7A	117.0	H15A—C15—H15B	109.5
C13—C8—C9	118.95 (17)	O4—C15—H15C	109.5
C13—C8—C7	122.56 (16)	H15A—C15—H15C	109.5
C9—C8—C7	118.5 (2)	H15B—C15—H15C	109.5
			
C4—C1—C2—C3	−0.5 (3)	C7—C8—C9—C10	178.20 (18)
C1—C2—C3—O1	0.2 (3)	C14—O3—C10—C9	8.3 (3)
C4—O1—C3—C2	0.2 (2)	C14—O3—C10—C11	−172.75 (17)
C2—C1—C4—O1	0.6 (2)	C8—C9—C10—O3	−178.34 (19)
C2—C1—C4—C5	179.8 (2)	C8—C9—C10—C11	2.8 (3)
C3—O1—C4—C1	−0.5 (2)	C15—O4—C11—C12	14.7 (3)
C3—O1—C4—C5	−179.81 (17)	C15—O4—C11—C10	−165.54 (18)
C1—C4—C5—O2	−179.5 (2)	O3—C10—C11—O4	−1.2 (2)
O1—C4—C5—O2	−0.4 (3)	C9—C10—C11—O4	177.80 (18)
C1—C4—C5—C6	0.5 (3)	O3—C10—C11—C12	178.59 (18)
O1—C4—C5—C6	179.55 (16)	C9—C10—C11—C12	−2.4 (3)
O2—C5—C6—C7	9.9 (3)	O4—C11—C12—C13	−179.89 (18)
C4—C5—C6—C7	−170.05 (18)	C10—C11—C12—C13	0.3 (3)
C5—C6—C7—C8	−178.99 (19)	C9—C8—C13—C12	−1.0 (3)
C6—C7—C8—C13	29.9 (3)	C7—C8—C13—C12	179.74 (19)
C6—C7—C8—C9	−149.4 (2)	C11—C12—C13—C8	1.4 (3)
C13—C8—C9—C10	−1.0 (3)		

### Hydrogen-bond geometry (Å, °)

**Table:**

*D*—H···*A*	*D*—H	H···*A*	*D*···*A*	*D*—H···*A*
C1—H1A···O2^i^	0.93	2.32	3.247 (3)	173
C3—H3A···O3^ii^	0.93	2.42	3.338 (3)	167
C7—H7A···O2	0.93	2.47	2.810 (3)	101
C14—H14B···Cg1^iii^	0.96	2.66	3.431 (2)	137

Symmetry codes: (i) *x*, *y*+1, *z*; (ii) *x*+1/2, *y*−1/2, *z*+1; (iii) −*x*+1/2, *y*−1/2, −*z*+2.
